# Clinical Outcomes of High-Dose Follitropin Delta in POSEIDON (Patient-Oriented Strategies Encompassing Individualized Oocyte Number) Group 4: An Exploratory Case Series on Follicular Efficiency and Embryo Development

**DOI:** 10.7759/cureus.92608

**Published:** 2025-09-18

**Authors:** Masato Kobanawa

**Affiliations:** 1 Gynecology, Kobanawa Clinic, Omitama City, JPN

**Keywords:** assisted reproductive technology, controlled ovarian stimulation, follicle-to-oocyte index, follitropin delta, gonadotropin-releasing hormone (gnrh) antagonist, individualized treatment, mean follicular output rate, poor responders

## Abstract

Background

Patients classified as Patient-Oriented Strategies Encompassing IndividualizeD Oocyte Number (POSEIDON) Group 4 represent a particularly challenging population in assisted reproductive technology (ART), due to advanced age and diminished ovarian reserve. While follitropin delta allows individualized dosing based on body weight and anti-Müllerian hormone (AMH) levels, its standard use in Japan is limited to 12 μg/day. The efficacy of higher off-label doses in this group remains insufficiently studied.

Methods

This retrospective case series included seven women aged ≥35 years with antral follicle count (AFC) <5 or AMH <1.2 ng/mL, treated at a single clinic between 2022 and 2024. All patients underwent controlled ovarian stimulation (COS) using a gonadotropin-releasing hormone (GnRH) antagonist protocol and received daily follitropin delta at doses of 15 μg. Key outcomes included follicular development, controlled ovarian stimulation (COS) and oocyte pick-up performance, embryological outcomes, and ovarian hyperstimulation syndrome (OHSS) incidence.

Results

The mean age of the participants was 44.0 years, and the mean AMH was 0.70 ng/mL. The average number of oocytes retrieved was 5.71, with all patients achieving follicle-to-oocyte index (FOI) ≥1.00 (mean 1.52). The mean follicular output rate (FORT) was 1.41. Good-quality blastocysts were obtained in five of seven cases (71.4%). None of the patients had OHSS despite relatively high gonadotropin exposure. No patient discontinued treatment, and the stimulation was well tolerated by all.

Conclusion

High-dose follitropin delta may represent a feasible and safe option for selected POSEIDON Group 4 patients. However, these findings are preliminary and derived from a small case series without a control group. Further prospective studies are required to confirm efficacy and long-term reproductive outcomes.

## Introduction

Controlled ovarian stimulation (COS) is a cornerstone of assisted reproductive technology (ART), aiming to recruit multiple follicles to maximize the number of oocytes available for fertilization and embryo development. However, a significant proportion of patients undergoing ART exhibit a suboptimal response to COS despite appropriate stimulation protocols. This has led to the development of refined classifications such as the Patient-Oriented Strategies Encompassing IndividualizeD Oocyte Number (POSEIDON) criteria, which stratify low-prognosis patients into four subgroups based on age, ovarian reserve markers, and prior ovarian response [1-4].

Patients categorized as POSEIDON Groups 1 to 4 represent a heterogeneous population unified by the shared challenge of achieving an adequate number of oocytes. Particularly, POSEIDON group 4 represents the most challenging category within the POSEIDON classification. It includes women aged 35 years or older with evidence of diminished ovarian reserve, defined as an antral follicle count (AFC) below 5 and/or anti-Müllerian hormone (AMH) levels below 1.2 ng/mL [1]. These patients are considered at high risk of poor ovarian response, with limited follicular recruitment despite stimulation, and reduced chances of obtaining a sufficient number of oocytes to achieve at least one euploid embryo. As a result, they are characterized by the lowest expected prognosis among the POSEIDON subgroups, and optimizing stimulation strategies for this population remains a major clinical challenge.

For these patients, optimizing follicular recruitment is critical, as even modest increases in oocyte yield can impact the cumulative live birth rate (CLBR) [5-7]. However, conventional strategies, such as increasing the dose of gonadotropins, have shown limited effectiveness in improving outcomes for poor responders [8]. Follitropin delta is a recombinant follicle-stimulating hormone (FSH) preparation that allows for individualized dosing based on a patient's body weight and AMH level [9]. It exhibits a distinct pharmacokinetic and pharmacodynamic profile, resulting in more predictable ovarian responses and a lower incidence of ovarian hyperstimulation syndrome (OHSS) [10].

In Japan, follitropin delta is approved for use at doses up to 12 μg/day. Nonetheless, emerging evidence, including the REKO15 trial, has demonstrated the safety and efficacy of higher daily doses (e.g., 15 μg) in oocyte donors [11], suggesting the potential utility of high-dose use in selected populations. To date, there is limited real-world data on the use of high-dose follitropin delta in POSEIDON-classified patients, particularly following treatment failures under insurance-covered standard protocols. In clinical practice, once such coverage ends, physicians may consider dose escalation as part of individualized strategies to optimize outcomes in patients with persistently low prognosis [12].

This case series reports on seven women classified as POSEIDON Group 4 who underwent COS with follitropin delta at daily doses of 15 µg. We analyzed their follicular development, oocyte yield, and embryo outcomes, with a particular focus on follicle-to-oocyte index (FOI), follicular output rate (FORT), and cumulative gonadotropin dose. The aim was to explore the feasibility and clinical utility of high-dose follitropin delta in this difficult-to-treat population, with the goal of informing whether higher-dose follitropin delta could be a viable individualized strategy in clinical practice.

## Materials and methods

This retrospective case series included seven patients treated at Kobanawa Clinic, Omitama City, Ibaraki, Japan, between 2022 and 2024. The study protocol, including off-label administration of follitropin delta, was reviewed and approved by the Kobanawa Clinic Ethics Review Board (approval number: 20250701). As a retrospective analysis using anonymized data, informed consent was obtained through an opt-out process. The study was conducted in accordance with the ethical standards of the institutional research committee and the 1964 Helsinki Declaration and its later amendments. All clinical and laboratory data were collected from the medical records at Kobanawa Clinic.

Study population

This study included patients who underwent COS using recombinant FSH (rFSH) monotherapy with follitropin delta (Rekovelle®; Ferring Pharma, Tokyo, Japan). Eligible patients were classified as POSEIDON Group 4, aged ≥35 years, with AFC <5 and/or AMH <1.2 ng/mL. Exclusion criteria included patients not meeting POSEIDON Group 4 definition, those receiving concomitant oral agents (e.g., clomiphene citrate or letrozole), and those treated with multiple injectable gonadotropin preparations or with HMG instead of rFSH monotherapy.

All consecutive patients who met these criteria, had demonstrated poor ovarian response in previous ART cycles, and were treated without concomitant use of other oral agents were included. All cycles were self-funded, as none of the patients were eligible for insurance coverage. In Japan, ART insurance coverage is restricted by age and cycle number; therefore, all included cases were self-funded after exhausting coverage [13,14].

Procedure

All patients were stimulated using a gonadotropin-releasing hormone (GnRH) antagonist protocol. Follitropin delta was administered subcutaneously once daily, starting on days 1-3 of menstruation. The initial daily dose was fixed at 15 μg and was not adjusted throughout stimulation. When the leading follicle reached approximately 14 mm in diameter, a GnRH antagonist (Ganirest® (ganirelix acetate)) was initiated at a dose of 0.25 mg/day and continued until the trigger day. Final oocyte maturation was triggered with 250 μg of choriogonadotropin alfa (Ovidrel®) when multiple leading follicles reached a diameter of 18-20 mm. Transvaginal oocyte retrieval was performed 34-36 hours after trigger injection. Insemination via conventional in vitro fertilization (IVF) or intracytoplasmic sperm injection (ICSI) was performed according to each patient’s clinical indications. ICSI was applied specifically in cases of male factor infertility or in patients with a history of fertilization failure in previous ART cycles. Fertilization was confirmed by the presence of two pronuclei, and embryos were cultured to the blastocyst stage (day 5 or 6). Blastocysts were graded according to Gardner’s classification [15], and high-quality blastocysts were defined as grade 3BB or higher. All good-quality blastocysts were vitrified and stored for future embryo transfer.

In Japan, the approved daily dose of follitropin delta is ≤12 µg. In this study, all patients received a daily dose of 15 µg, which constitutes off-label use. The starting and ongoing daily dose of follitropin delta was fixed at 15 µg without further adjustment during stimulation. Dose escalation or reduction was not permitted in this protocol. The decision to use an increased dose was made after patients had completed all insurance-covered treatment cycles and were undergoing self-funded ART. Prior to treatment, the rationale and regulatory context were explained directly to each patient during face-to-face consultation, and all patients provided written informed consent for the off-label administration of follitropin delta above 12 µg/day, after being thoroughly informed about the rationale, potential risks, and regulatory status of this dosing regimen.

Outcomes and measurements

Key outcomes included the number and size distribution of follicles at trigger, number of oocytes retrieved, number of two-pronuclear (2PN) zygotes, number of good-quality blastocysts, and the incidence of OHSS. OHSS was defined according to the modified Golan classification, with Grade 1 or higher (presence of abdominal distension) considered as the threshold for diagnosis [16]. In addition, patients were actively questioned about potential adverse symptoms, including abdominal bloating, injection-site discomfort, headache, and fatigue, to ensure systematic assessment rather than relying solely on volunteered reports. All patients were specifically evaluated for signs and symptoms of OHSS at two to three days and again at seven days after oocyte retrieval.

FOI was calculated as the number of oocytes retrieved divided by the AFC [17], and FORT was defined as the number of follicles ≥16 mm on the day of trigger divided by the AFC [18]. AFC was measured by transvaginal ultrasonography during the early follicular phase (cycle day 2-4), counting follicles measuring 2-10 mm in both ovaries by the same physician [19]. In addition, the total dose of gonadotropins administered during stimulation and the serum hormone profiles, including estradiol (E2), progesterone (P4), and luteinizing hormone (LH), were evaluated.

## Results

A total of seven patients meeting the POSEIDON Group 4 criteria (age ≥35 years and AFC <5 or AMH＜1.2) were included in this case series. The mean age was 44 years (range, 41-46), reflecting a very advanced-age cohort, and the median AFC was 4. All patients had low ovarian reserve, with an average AMH level of 0.70 ng/mL (range, 0.23-1.38) (Table 1).

**Table 1:** Baseline characteristics of the seven patients included in the case series. AFC, antral follicle count; AMH, anti-Müllerian hormone; FSH, follicle-stimulating hormone; E2: estradiol; LH: luteinizing hormone

Case number	Age (years)	Body weight (kg)	Previous retrieval cycles	AFC	AMH, ng/ml	Basal FSH, IU/L	Basal E2, pg/ml	Basal LH, IU/L
1	44	74.1	3	4	1.38	5.36	74.48	4.69
2	44	78.6	2	4	0.99	7.01	32.1	1.91
3	45	46.6	3	4	0.78	5.9	73.23	6.2
4	41	78.4	4	4	0.43	7.12	58.31	3.11
5	45	55.9	1	2	0.32	9.06	51.52	9.68
6	43	64	3	5	0.23	1.66	118.6	1.71
7	46	65.9	1	3	0.8	5.72	8.14	4.54
Overall Mean Values	44	66.21	2.43	3.71	0.70	5.98	59.48	4.55
Overall Median Values	44	65.90	3.00	4.00	0.78	5.90	58.31	4.54

The median duration of stimulation was 14 days (range, 11-19), and the mean total gonadotropin dose administered was 212.14 µg (range, 165-285 µg). All patients received only a daily dose of 15 µg throughout the COS. The mean number of oocytes retrieved was 5.71 (range, 2-10). All patients achieved an FOI ≥1.00 (mean 1.52, range 1.00-2.50). While this may appear relatively high, such values should be interpreted cautiously, given the small sample size and the advanced age/low ovarian reserve of the cohort. FORT also demonstrated a modest response, with a mean of 1.41 (range, 0.50-2.33). Good-quality blastocysts were obtained in five of the seven cases (71.43%), with one or two blastocysts formed per patient. Notably, these outcomes were achieved even in patients with a history of multiple prior retrieval cycles (median = 3), underscoring the potential benefit of high-dose follitropin delta stimulation. Importantly, no cases of OHSS were reported (Table 2).

**Table 2:** Descriptive statistics of stimulation outcomes, follicular response, and embryological parameters (N = 7) OHSS: ovarian hyperstimulation syndrome

Variables	Mean	SD	Minimum	25th percentile	50th percentile	75th percentile	Maximum
Stimulation period (days)	14.14	2.67	11	12.5	14	15	19
Follicle count ≧14mm on days 6-8 of stimulation	2.43	1.51	0	1.5	3	3.5	4
Minimum follicle diameter on days 6-8 of stimulation (mm)	9.29	3.15	5	8	8	11	14
Maximum follicle diameter on days 6-8 of stimulation (mm)	14	5.35	5	12	15	16	22
Total gonadotropin dose (μg)	212.14	40.09	165	187.5	210	225	285
E2 level on the day of trigger (pg/ml)	1806.7	1003.03	544.9	1130	1728	2295	3524
P4 level on the day of trigger (ng/ml)	1.53	0.99	0.26	0.91	1.4	2.17	2.91
Follicle count ≧14 mm on the day of trigger	6.71	2.93	2	5.5	7	8	11
Follicle count ≧16 mm on the day of trigger	5.29	2.43	1	4	6	7	8
Follicle count ≧18 mm on the day of trigger	4.29	1.98	1	3.5	4	5.5	7
Follicle count ≧20 mm on the day of trigger	2.86	2.12	0	1	4	4.5	5
Number of oocytes retrieved	5.71	2.81	2	4.5	5	7	10
Number of two-pronuclei (2PN) zygotes	3.14	2.34	0	1.5	4	4	7
Number of good-quality blastocysts	1	0.82	0	0.5	1	1.5	2
Incidence of OHSS (%)	0	0	0	0	0	0	0
Follicular output rate (FORT)	1.41	0.67	0.5	0.9	1.5	1.88	2.33
Follicle-to-oocyte index (FOI)	1.52	0.63	1	1	1.25	1.96	2.5

Moreover, no patients experienced adverse events such as headache, abdominal bloating, injection-site reactions, or mood changes. Despite the relatively high dose and prolonged duration of gonadotropin use, all patients tolerated the protocol well, and no cycle was cancelled due to inadequate response or adverse effects (Table 3). 

**Table 3:** Incidence of adverse events observed during controlled ovarian stimulation with high-dose follitropin delta (15 µg/day) in POSEIDON Group 4 patients (N = 7)

Adverse event	Incidence, n (%)
Ovarian hyperstimulation syndrome (OHSS)	0 (0)
Headache	0 (0)
Abdominal bloating	0 (0)
Injection-site reaction	0 (0)
Mood changes / irritability	0 (0)
Cycle cancellation	0 (0)

Detailed individual-level data for all seven cases, including stimulation parameters, hormonal profiles, follicular development, and embryological outcomes, are provided in Table 4. Stimulation lasted 11-19 days with total doses of 165-285 µg, yielding 2-10 oocytes. Good-quality blastocysts were obtained in four cases, and no OHSS was observed.

**Table 4:** Comprehensive individual data of the seven cases, including stimulation details, follicular response, hormonal profiles, embryological outcomes, and key performance indicators E2, estradiol; P4, progesterone; 2PN, two-pronuclei; OHSS, ovarian hyperstimulation syndrome; FORT, follicular output rate; FOI, follicle-to-oocyte index; ICSI, intracytoplasmic sperm injection; c-IVF, conventional in vitro fertilization

Case number	Stimulation period (days)	Number of follicles≧14mm on days 6–8 of stimulation	Minimum follicle diameter on days 6–8 of stimulation (mm)	Maximum follicle diameter on days 6–8 of stimulation (mm)	Total gonadotropin dose (mcg)	E2 level on the day of trigger (pg/ml)	P4 level on the day of trigger (ng/ml)	Follicle count ≥14 mm on the day of trigger	Follicle count ≥16 mm on the day of trigger	Follicle count ≥18 mm on the day of trigger	Follicle count ≥20 mm on the day of trigger	Number of oocytes retrieved	Fertilization methods	Number of 2PN zygotes	Number of good-quality blastocysts	Incidence of OHSS (%)	FORT	FOI
1	14	4	8	15	210	1118	2.91	11	7	7	4	10	ICSI	2	1	0	1.75	2.50
2	11	3	9	17	165	2514	0.972	7	6	5	5	4	c-IVF	4	1	0	1.50	1.00
3	12	1	8	14	180	3524	2.79	8	8	6	5	9	c-IVF	7	2	0	2.00	2.25
4	13	4	14	22	195	1142	0.852	4	4	4	1	5	ICSI	1	0	0	1.00	1.25
5	14	2	13	15	210	544.9	0.255	2	1	1	1	2	c-IVF	0	0	0	0.50	1.00
6	19	0	5	5	285	2076	1.55	7	4	4	4	5	c-IVF	4	1	0	0.80	1.00
7	16	3	8	10	240	1728	1.4	8	7	3	0	5	c-IVF	4	2	0	2.33	1.67

## Discussion

In Japan, government insurance coverage for ART is limited by age and number of attempts (three embryo transfer attempts in women aged 40-42 and six attempts in women under 40 years of age). Patients who exceed these limits must proceed with self-financed treatment [13,14]. This regulatory context explains why all patients in our study underwent self-funded cycles and highlights how healthcare policy influences clinical decision-making and off-label use of higher gonadotropin doses. A notable strength of this study is its real-world, post-insurance setting. In Japan, patients who have exhausted insurance-covered ART cycles often face limited therapeutic options. Our cohort reflects this clinical reality, where individualized strategies such as off-label dose escalation may provide a meaningful alternative.

In this case series, we evaluated the clinical efficacy and safety of high-dose follitropin delta (15μg/day) in patients classified as low prognosis according to the POSEIDON criteria. This population represents a diverse but clinically challenging subset of patients undergoing ART, characterized by impaired ovarian response and/or reduced oocyte competence. The POSEIDON stratification offers a clinically relevant framework by subdividing low responders based on age, ovarian reserve markers (AMH and AFC), and previous response to stimulation [1]. While Groups 3 and 4 represent patients with diminished ovarian reserve (AMH <1.2 ng/mL or AFC <5), Groups 1 and 2 include women with normal ovarian reserve but an unexpected poor or suboptimal response in prior stimulation. These distinctions highlight the need for personalized stimulation strategies tailored to each subgroup's pathophysiology [3].

Follitropin delta is a recombinant FSH designed for individualized dosing based on body weight and AMH levels. The drug’s pharmacokinetic and pharmacodynamic properties allow for consistent FSH exposure and predictable ovarian response [20]. However, in Japan, its clinical use is limited to ≤12 μg/day. Our study explores off-label use beyond this limit, assessing cycles with 15μg/day dosing among POSEIDON Group 4 patients, classified as low prognosis. Our study showed the mean FORT was 1.41 and the mean FOI was 1.52. Both clearly exceed the minimum expected thresholds of ≥ 40% for FORT and ≥ 50% for FOI, as defined by the recent consensus guidelines [21].

Our findings suggest that dose escalation with follitropin delta may achieve enough follicular recruitment and blastocyst development for POSEIDON Groups 4, with no OHSS reported. This aligns with findings from the REKO15 trial, which demonstrated that a daily dose of 15 μg of follitropin delta provided a comparable ovarian response to 225 IU/day of follitropin alfa in normo-responders undergoing progestin-primed ovarian stimulation [11]. The trial also suggested that follitropin delta could reduce total gonadotropin consumption and stimulation duration while maintaining a high level of patient satisfaction. Our study demonstrated the feasibility and safety of 15 μg/day follitropin delta in the most challenging population, patients with low prognosis classified as POSEIDON Group 4. By contrast, the REKO15 trial was conducted in young oocyte donors with high ovarian reserve, a biologically distinct group [11]. Although REKO15 provided reassuring safety data in donors, whether these findings are applicable to poor responders remains uncertain.

In our study, patients received follitropin delta monotherapy, isolating its effect and avoiding confounding from other agents such as human menopausal gonadotropin (hMG) or clomiphene. This study specifically addresses POSEIDON Group 4, the subgroup with the poorest prognosis due to advanced age and diminished ovarian reserve. In this context, our results raise the hypothesis that the conventional ‘dose-ceiling’ concept for gonadotropins may not fully apply. However, these findings remain exploratory and require validation in larger, controlled studies before firm clinical conclusions can be drawn. Previous comparative studies have indicated that daily doses of 8-12 μg of follitropin delta result in a similar oocyte yield to 150 IU of follitropin alfa [22,23]. Based on this equivalence, patients who require >225-300 IU of daily gonadotropin, commonly observed among poor or suboptimal responders, may receive an insufficient stimulus under the standard follitropin delta algorithm. In this context, our demonstration of the efficacy and safety of 15μg/day dosing provides a valuable option for enhancing follicular recruitment in those patients who might otherwise experience inadequate response under standard dosing paradigms. Furthermore, follitropin delta has lower clearance rates, leading to greater exposure and greater pharmacodynamic response than follitropin alfa [24]. Therefore, follitropin delta may exert a stronger stimulatory effect on follicular recruitment and development.

Across the seven cases, higher FOI and FORT values tended to occur in patients with relatively higher baseline AMH, whereas age showed no clear correlation with response parameters. Good-quality blastocysts were observed even in patients with multiple previous retrieval failures, suggesting that dose escalation may sometimes overcome prior poor responses. Moreover, the precision of delta’s mcg-based dosing and reduced variability in FSH bioactivity compared to IU-based preparations may offer a particular advantage in patients where even one additional mature oocyte can influence cumulative live birth outcomes. Previous studies have shown that the number of oocytes retrieved is directly associated with CLBR [25-28]. For patients with low prognosis, achieving this threshold is often unrealistic, but maximizing oocyte yield within safety limits remains an essential strategy.

This study’s strengths include a well-defined low-prognosis population, use of monotherapy to isolate the drug effect, and a focus on a practical clinical setting after insurance-covered cycles have ended. However, this study has several limitations. First, the sample size was small (n=7), and the retrospective design limits the generalizability of the findings. Second, there was no control group for comparison, making it difficult to directly attribute outcomes to high-dose follitropin delta rather than patient-specific variability. Third, clinical outcomes such as pregnancy rates and live birth rates were not assessed; therefore, the implications of improved follicular recruitment and blastocyst development on ultimate reproductive success remain uncertain. Fourth, embryo euploidy status was not evaluated, which is particularly relevant in older patients with diminished ovarian reserve. Finally, the use of follitropin delta at 15 µg/day represents off-label administration in Japan. Although conducted with patient consent and ethics committee approval, this limits the direct applicability of the results in broader regulatory contexts. Future prospective studies with larger cohorts, inclusion of live birth outcomes, and comparative analyses against standard protocols are warranted to validate the clinical utility of this strategy. This study does not establish efficacy but should be regarded as signal-generating, highlighting the feasibility and safety of high-dose follitropin delta in this difficult-to-treat population. Owing to the small sample size, retrospective design, and lack of a control group, the results should be interpreted as preliminary and hypothesis-generating rather than definitive evidence. Within this uniformly low-prognosis Group 4 cohort, modest differences in baseline reserve markers such as AMH appeared to influence follicular recruitment and embryo development. Nonetheless, the variability observed, including single-patient extremes, underscores the anecdotal nature of these findings and the need for validation in larger studies.

## Conclusions

This exploratory case series suggests that high-dose follitropin delta may be a feasible and safe option for selected patients classified as POSEIDON Group 4. Our findings highlight the potential clinical utility of individualized dose escalation in this difficult-to-treat population, warranting formal evaluation in prospective studies. Future research should focus on defining the optimal dosing strategies, assessing cost-effectiveness, and clarifying long-term reproductive outcomes to improve care for women with the lowest reproductive prognosis.
